# Lamp1 Increases the Efficiency of Lassa Virus Infection by Promoting Fusion in Less Acidic Endosomal Compartments

**DOI:** 10.1128/mBio.01818-17

**Published:** 2018-01-02

**Authors:** Christine E. Hulseberg, Lucie Fénéant, Katarzyna M. Szymańska, Judith M. White

**Affiliations:** a Department of Microbiology, University of Virginia, Charlottesville, Virginia, USA; b Department of Cell Biology, University of Virginia, Charlottesville, Virginia, USA; Columbia University Medical College

**Keywords:** CD63, Lujo virus, arenavirus, endosomes, fusion trigger, intracellular receptor, Lassa fever, low pH, lymphocytic choriomeningitis virus, virus entry

## Abstract

Lassa virus (LASV) is an arenavirus whose entry into host cells is mediated by a glycoprotein complex (GPC) comprised of a receptor binding subunit, GP1, a fusogenic transmembrane subunit, GP2, and a stable signal peptide. After receptor-mediated internalization, arenaviruses converge in the endocytic pathway, where they are thought to undergo low-pH-triggered, GPC-mediated fusion with a late endosome membrane. A unique feature of LASV entry is a pH-dependent switch from a primary cell surface receptor (α-dystroglycan) to an endosomal receptor, lysosomal-associated membrane protein (Lamp1). Despite evidence that the interaction between LASV GP1 and Lamp1 is critical, the function of Lamp1 in promoting LASV infection remains poorly characterized. Here we used wild-type (WT) and Lamp1 knockout (KO) cells to show that Lamp1 increases the efficiency of, but is not absolutely required for, LASV entry and infection. We then used cell-cell and pseudovirus-cell surface fusion assays to demonstrate that LASV GPC-mediated fusion occurs at a significantly higher pH when Lamp1 is present compared to when Lamp1 is missing. Correspondingly, we found that LASV entry occurs through less acidic endosomes in WT (Lamp1-positive) versus Lamp1 KO cells. We propose that, by elevating the pH threshold for fusion, Lamp1 allows LASV particles to exit the endocytic pathway before they encounter an increasingly acidic and harsh proteolytic environment, which could inactivate a significant percentage of incoming viruses. In this manner Lamp1 increases the overall efficiency of LASV entry and infection.

## IMPORTANCE

Lassa virus is the most clinically important member of the *Arenaviridae*, a family that includes six additional biosafety level 4 (BSL4) hemorrhagic fever viruses. The lack of specific antiviral therapies for Lassa fever drives an urgent need to identify druggable targets, and interventions that block infection at the entry stage are particularly attractive. Lassa virus is only the second virus known to employ an intracellular receptor, the first being Ebola virus. Here we show that interaction with its intracellular receptor, Lamp1, enhances and upwardly shifts the pH dependence of fusion and consistently permits Lassa virus entry into cells through less acidic endosomes. We propose that in this manner, Lamp1 increases the overall efficiency of Lassa virus infection.

## INTRODUCTION

Lassa virus (LASV) is the most clinically important member of the *Arenaviridae*, a diverse family of enveloped, negative-sense RNA viruses, which currently includes seven recognized hemorrhagic fever viruses (1). Infections in humans typically involve inhalation of the excreta of rodents, which are the natural reservoirs of the viruses, or ingestion of contaminated food or water (2, 3). Arenaviruses are classified into two groups according to their phylogenetic relatedness and the geographic range of their respective rodent carriers: New World arenaviruses are limited to the Americas, and Old World arenaviruses, which include LASV, are generally confined to Africa (4). Lymphocytic choriomeningitis virus (LCMV), an Old World arenavirus with worldwide distribution, is of particular note because it has long served as a prototypical arenavirus and is among the best studied of all viruses.

As with other Old World arenaviruses, LASV particles use trimeric glycoprotein spikes (5, 6) on their surface to engage the alpha subunit of dystroglycan (α-DG), its primary cell surface receptor (7). Upon binding to α-DG, LASV particles are internalized into compartments in the endocytic pathway. The acidified environment within maturing endosomes eventually triggers fusion between the viral and endosomal membranes, allowing the viral genome to be released through the resulting fusion pore into the cytoplasm. This membrane fusion event is mediated by the viral glycoprotein complex (GPC), which is comprised of a receptor binding subunit, GP1, a fusogenic transmembrane subunit, GP2, and a stable signal peptide. As the pH of the endocytic compartment decreases, GP1 dissociates from the complex, triggering major reorganizational changes in GP2 and uncovering the hydrophobic fusion loop that drives membrane fusion (8–10).

An interesting feature of LASV is that it employs a second, intracellular receptor. En route in the endocytic pathway, the GP1 subunit undergoes a pH-dependent switch from the α-DG surface receptor to its endosomal receptor, Lamp1 (9, 11). This pairing of LASV GP1 with Lamp1 is only the second example of a virus using an intracellular receptor, the first being the use of Niemann-Pick C1 (NPC1) by the GP1 of Ebola virus and other filoviruses (12–14). While these interactions are now well documented (11, 15, 16), the precise manner(s) in which Lamp1 and NPC1 promote viral entry remains unknown.

In this study, we explored the role of Lamp1 in LASV fusion and entry. We first found that a low level of Lamp1 supports robust entry and that entry can even occur, albeit attenuated, in cells lacking Lamp1. We next showed that Lamp1 upwardly shifts the pH dependence of LASV GPC-mediated fusion from its unusually low optimum of pH ≤ 4 (17, 18) to the higher, more physiological range found within the endocytic pathway. Consistently, we found that entry of LASV GPC pseudoviruses occurs in less acidic endosomes in cells containing versus cells lacking Lamp1. Taken together, we propose that Lamp1 increases the overall efficiency of LASV entry and infection by promoting fusion in a more hospitable, less acidic endosomal compartment.

## RESULTS

### A low level of Lamp1 supports robust LASV GPC-mediated infection.

**FIG 1:**
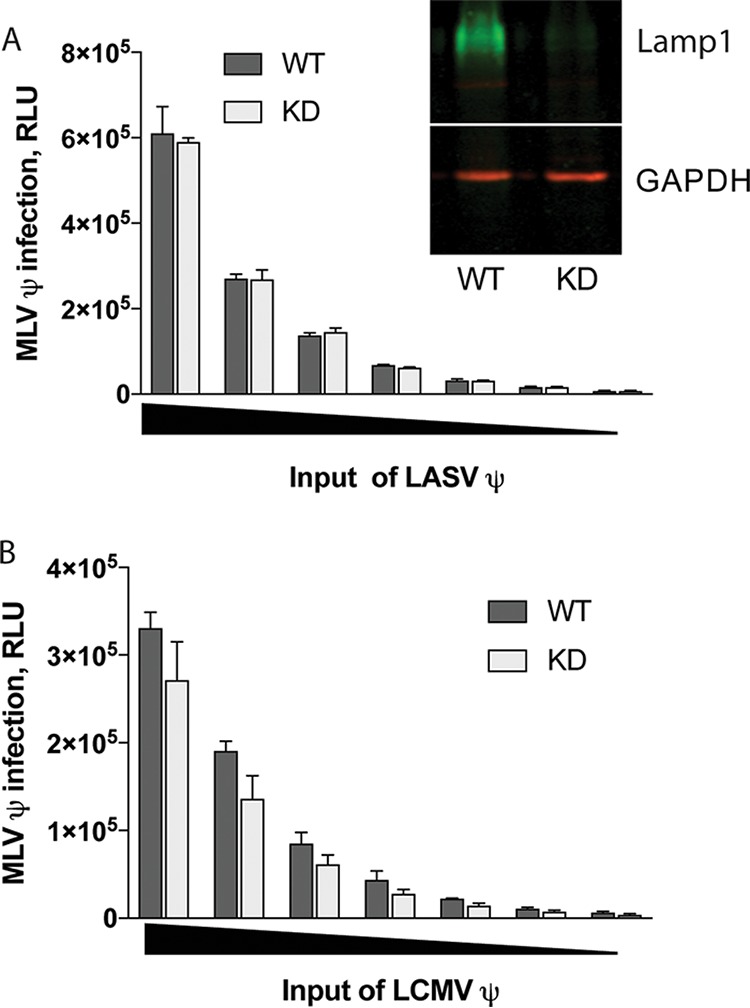
Knockdown of Lamp1 does not suppress LASV pseudovirus infection. The effect of Lamp1 deficiency on LASV (A) and LCMV (B) pseudovirus (denoted by ψ) infection over a range of pseudoviral inputs (indicated by a black triangle on the abscissa) was evaluated based on expression of the luciferase reporter. Lamp1-knockdown (KD) cells express 17.1% ± 7.8% WT levels of Lamp1 (*n* = 7) (inset in panel A). Each data point is the average of triplicate measurements from one representative experiment (performed five times with similar results). Error bars indicate standard deviation (SD). KD values did not significantly differ from WT values in any data point by unpaired, two-tailed *t* test.

Lamp1 was recently reported to serve as the intracellular receptor for LASV (9). To begin to explore the role of Lamp1, we first generated a stable line of 293T cells in which Lamp1 expression was strongly reduced using a lentivirus encoding a short hairpin RNA (shRNA) to knock down (KD) Lamp1 expression. This knockdown reduced Lamp1 expression to ~15% relative to expression in WT cells (Fig. 1A, inset and legend). Nonetheless, Lamp1 KD cells and wild-type (WT) cells were equally susceptible to infection with MLV pseudoviruses bearing LASV GPC (and encoding luciferase) at all inputs of virus tested (Fig. 1A). Since LCMV GPC does not interact with Lamp1, LCMV infections were, as expected, unaffected by decreased Lamp1 expression (Fig. 1B). This finding (Fig. 1A) suggested that the residual Lamp1 expression in Lamp1 KD cells is sufficient to fully support efficient LASV GPC-mediated infection.

### The absence of Lamp1 reduces, but does not eliminate, LASV GPC-mediated entry and infection.

**FIG 2:**
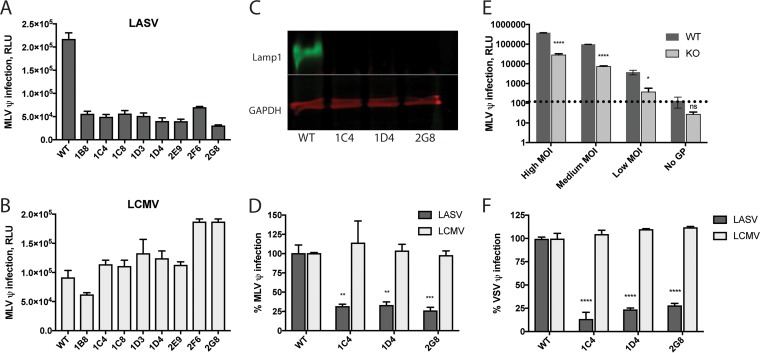
Knockout of Lamp1 reduces but does not abolish LASV GPC-mediated infection. Eight Lamp1 KO clones (see Materials and Methods) were screened for infection with LASV (A) and LCMV (B) MLV pseudoviruses. Data represent average luminescence units ± SD, measured in triplicate, from one experiment. (Two additional experiments with similar results were performed.) The level of LASV infection across these eight Lamp1 KO clones was 22% ± 5.3% of that seen in WT cells. (C) Representative blot (out of five similar blots) of lysates from three KO clones showing that Lamp1 was not detectable. (D) The above clonal KO lines were infected in quadruplicate with a moderate input (titer determined for a signal of ~100,000 relative light units [RLU] in WT cells) of either LASV or LCMV MLV luciferase pseudoviruses. Data were normalized to maximal signal from WT cells, and statistical significance was calculated by comparing the percentage of infection in KO cells against the percentage of infection in WT cells using a two-way analysis of variance (ANOVA). Error bars represent SD. **, *P* < 0.01; ***, *P* < 0.001. (E) One representative clone (2G8) was assayed in triplicate for infection with high, medium, and low input levels of LASV GPC pseudoviruses. Pseudoviruses lacking glycoprotein (“No GP”) were used to establish a background signal, indicated by a dashed line. Error bars represent SD. *, *P* < 0.05, ****, *P* < 0.0001, and ns, not significant, based on multiple unpaired, two-tailed *t* tests. The experiment was repeated twice with similar results. (F) The same clonal KO lines tested in panel D were infected with LASV and LCMV VSV pseudoviruses encoding GFP, and the titer was determined for 40 to 70% infection in WT cells. Signal from the “No GP” control pseudoviruses was subtracted from all measurements. As in panel D, infection signal from triplicate samples was normalized to WT cells and statistical analysis was applied to the efficiency of infection in KO cells compared to WT cells. The experiment was repeated two times with similar results.

To test the effects of a complete loss of Lamp1 on LASV entry and infection, we generated Lamp1 knockout (KO) cell lines using clustered regularly interspaced short palindromic repeats with Cas9 (CRISPR/Cas9) gene editing (see Fig. S1 in the supplemental material). An initial assessment of LASV murine leukemia virus (MLV) pseudovirus infection in eight clonal KO cell lines revealed that infection occurred but was reduced to ~20% of the efficiency in WT cells (Fig. 2A). In contrast, and as expected, LCMV MLV pseudoviruses were as infectious in the KO cell lines as they were in the parental WT cells (Fig. 2B). Three of these KO cell lines—all of which demonstrated undetectable levels of Lamp1 via Western blotting (Fig. 2C)—were selected for further testing. We again found that these KO cell lines were susceptible to LASV MLV pseudovirus infection at ~15 to 30% of the level seen in WT cells (Fig. 2D). Notably, this low level of infection was seen across a wide range of pseudovirus inputs (Fig. 2E). To assess this finding with a different pseudovirus system, we performed the analysis again using vesicular stomatitis virus (VSV) pseudoviruses bearing LASV or LCMV GPC and encoding a green fluorescent protein (GFP) reporter (Fig. 2F). In agreement with the findings using MLV pseudoviruses, we found that LASV VSV pseudoviruses infect Lamp1 KO cells at ~15 to 30% of the efficiency in WT cells, while LCMV VSV pseudoviruses infected KO cells as efficiently as WT cells.

10.1128/mBio.01818-17.1 FIG S1  Overview of workflow for generating and validating Lamp1 KO 293T cells. Four gRNAs targeting early Lamp1 exons were cloned into the pX330-U6 vector (dual Cas9-gRNA scaffold). 293T cells were then separately transfected with each of the gRNAs (and one population transfected with all four gRNAs) and lysed 1 week later for comparison of total Lamp1 levels via Western blotting. The cell population transfected with gRNA 1 (indicated by black triangle) was stained with Lamp1 antibody and AF-488 and then subjected to negative selection by FACS. After allowing for expansion of singly sorted cells, clonal cell lines were seeded onto 96-well plates, permeabilized, and screened for Lamp1 expression via in-cell Western assay. Cells from Lamp1-negative clones were lysed and further confirmed for null Lamp1 expression by Western blotting. Two of these clones and parental cells were then subjected to genomic sequencing around the PAM site. Note the mixed sequence for the 2G8 clone suggests that the alleles were modified differently; however, both alleles are disrupted relative to WT sequence. Download FIG S1, TIF file, 1.7 MB. Copyright © 2018 Hulseberg et al. 2018 Hulseberg et al. This content is distributed under the terms of the Creative Commons Attribution 4.0 International license.

**FIG 3:**
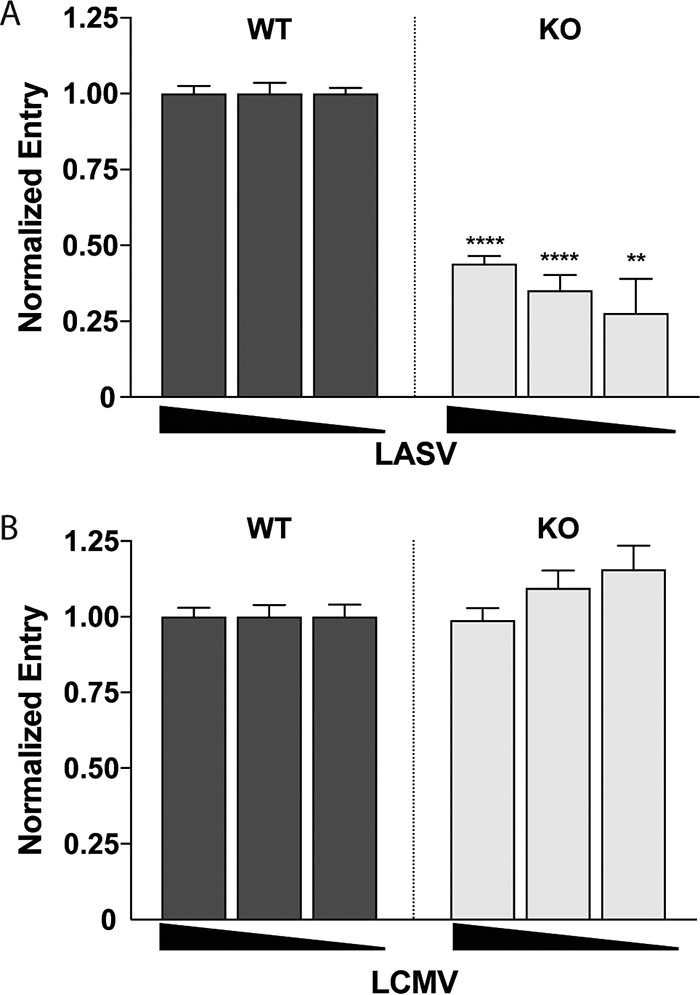
Knockout of Lamp1 reduces, but does not eliminate, LASV GPC-mediated entry. Lamp1-dependent entry was assayed by infecting WT and KO cells in triplicate with high, medium, and low inputs of (A) LASV and (B) LCMV MLV-βlaM pseudoviruses. Fluorescent signals (indicating cleavage of βlaM upon entry into the cytoplasm) from infected KO cells were normalized to those from WT cells at each input. Background signal from uninfected control cells loaded with βlaM substrate was subtracted from all data points. A negative-control infection using “No GP” pseudoviruses (not shown) generated a fluorescent signal roughly equivalent to the substrate-only background signal. Data show the average of normalized triplicate fluorescence measurements ± SD from a single representative experiment. The experiment was performed three times with similar results. **, *P* < 0.01, and ****, *P* < 0.0001, based on unpaired, two-tailed *t* test.

The findings in Fig. 2 (using two different types of pseudoviruses whose only common feature is expression of LASV GPC) suggested that GPC-mediated entry is reduced, but not abolished, in these Lamp1 KO cells. To test this proposal, we used a complementary approach and infected cells with pseudoviruses carrying an MLV-Gag-β-lactamase (βlaM) chimeric protein. Upon entering the cytoplasm, Gag-βlaM cleaves the CCF2-AM substrate (which is loaded into cells shortly after infection), and the resulting change in fluorescence of the product from green to blue provides a sensitive readout for viral entry (19–22). We infected WT and Lamp1 KO cells with a range of MLV-Gag-βlaM pseudovirus inputs bearing either LASV or LCMV GPC. As seen in Fig. 3, entry mediated by LASV GPC occurred in Lamp1 KO cells at ~25 to 35% of the level seen in WT cells. In contrast, and as expected, entry of pseudoviruses bearing LCMV GPC occurred at roughly the same level in WT and KO cells.

The findings presented in Fig. 1 to 3 suggest that, in our system, Lamp1 increases the efficiency of, but is not absolutely required for, LASV GPC-mediated entry and infection. In Lamp1 KO 293T cells, entry and infection by both MLV and VSV pseudovirus particles expressing LASV GPC occur at ~20 to 30% of the level seen in WT cells. We note that the requirement for Lamp1 appears more stringent using authentic LASV in different cells (9). The fusion-enhancing effect of Lamp1 is also specific, as it is not seen for LCMV GPC-mediated entry or infection. Moreover, results from our KD analysis indicate that a low level of Lamp1 (~15% of WT) in 293T cells suffices for efficient LASV GPC-mediated entry and infection.

### Lamp1 increases the extent of, and raises the pH threshold for, LASV GPC-mediated fusion.

**FIG 4:**
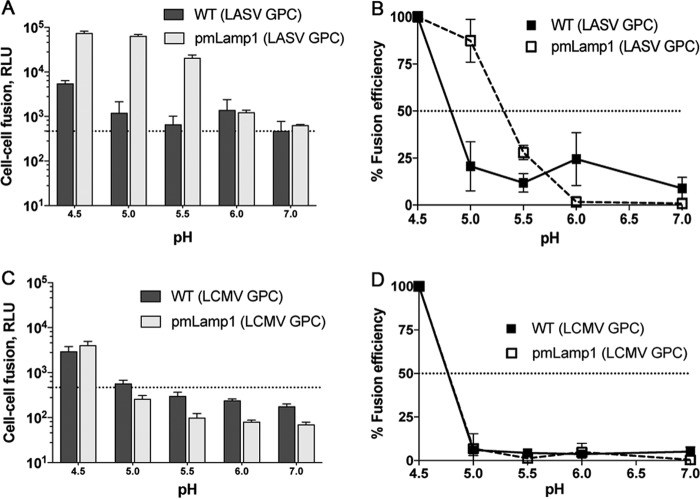
Lamp1 increases the extent and raises the pH threshold of LASV GPC-mediated fusion. In panels A and C, luminescence shows the extent of cell-cell fusion with WT (dark boxes) or pmLamp1 (light boxes) cells for LASV (A) and LCMV (C). Data represent RLU ± SD from the average of triplicate measurements. Dashed lines in panels A and C indicate background signal. In panels B and D, the data were normalized to fusion at pH 4.5 and replotted to show the corresponding pH dependence of cell-cell fusion for LASV (B) and LCMV (D). Dashed lines in panels B and D indicate 50% fusion efficiency. The LASV experiment was performed two additional times with similar results. Error bars represent the average ± SD of normalized values.

**FIG 5:**
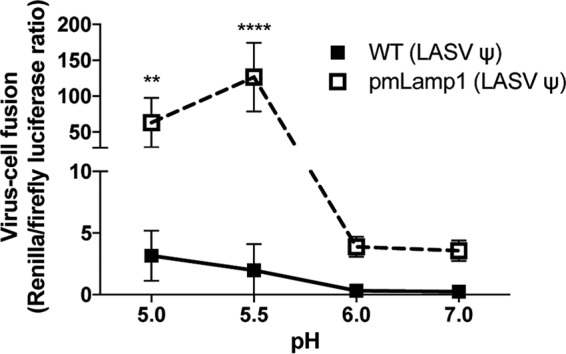
The extent and pH dependence of LASV pseudovirus (ψ) fusion with the cell surface in the presence and absence of pmLamp1. LASV VSV pseudoviruses were bound to precooled, untransfected WT or pmLamp1-expressing COS-7 cells. The cells were pulsed at the indicated pH for 5 min at 37°C, reneutralized, and then treated with 40 mM NH_4_Cl to raise endosomal pH. After 24 h, cells were lysed and assessed for viral fusion with the plasma membrane using the ratio of *Renilla* luciferase activity (virus replication) over firefly luciferase activity (number of cells). Data are from a single experiment and represent average RLU ± SD of sextuplicate measurements. Statistical significance of fusion with WT versus pmLamp1 cells was demonstrated at pH 5.0 and 5.5 using multiple unpaired *t* tests (**, *P* < 0.01; ****, *P* < 0.0001). A Grubbs’ test permitted removal of an outlier (*Z* = 1.7715) from a measurement of fusion with pmLamp1 cells at pH 7.0. The experiment was repeated a second time with virtually identical results.

**FIG 6:**
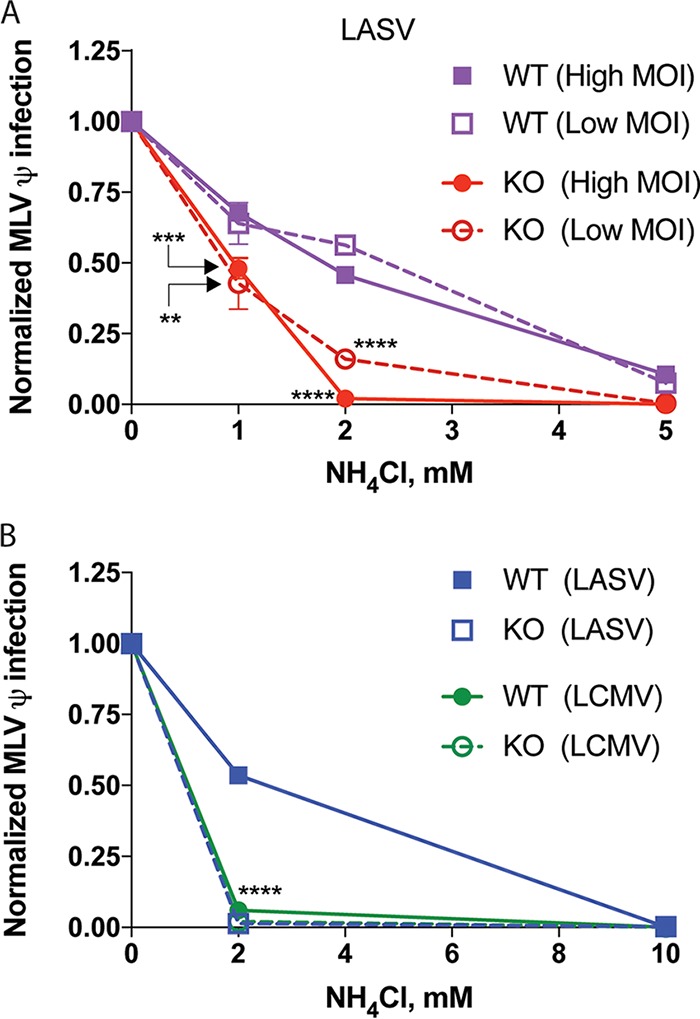
LASV, but not LCMV, GPC-mediated infection is more sensitive to NH_4_Cl in cells lacking Lamp1. Lamp1 WT and KO cells were pretreated with NH_4_Cl at the indicated concentrations. WT (purple in panel A) or KO (red in panel A) cells were then infected with (A) LASV MLV pseudoviruses at high input (titer determined for ~150,000-RLU signal in mock-treated WT cells [closed symbols]) or low input (titer determined for ~50,000-RLU signal [open symbols) or (B) LASV (blue in panel B) and LCMV (green in B) MLV pseudoviruses at a single input (titer determined for ~100,000 RLU signal in mock-treated WT cells). At 24 hpi, cells were lysed and analyzed for firefly luciferase activity. Infection signal was normalized to mock-treated cells. At 1 and 2 mM NH_4_Cl concentrations in panel A, KO infection was compared to WT infection at either high or low multiplicity of infection (MOI) using an unpaired, two-tailed *t* test. In panel B, a one-way ANOVA was used to compare LCMV-infected cells (both WT and KO) and LASV-infected KO cells, to LASV-infected WT cells at 2 mM NH_4_Cl treatment. **, *P* < 0.01; ***, *P* < 0.001; and ****, *P* < 0.0001. Data are from a single experiment that was performed two additional times with similar results.

**FIG 7:**
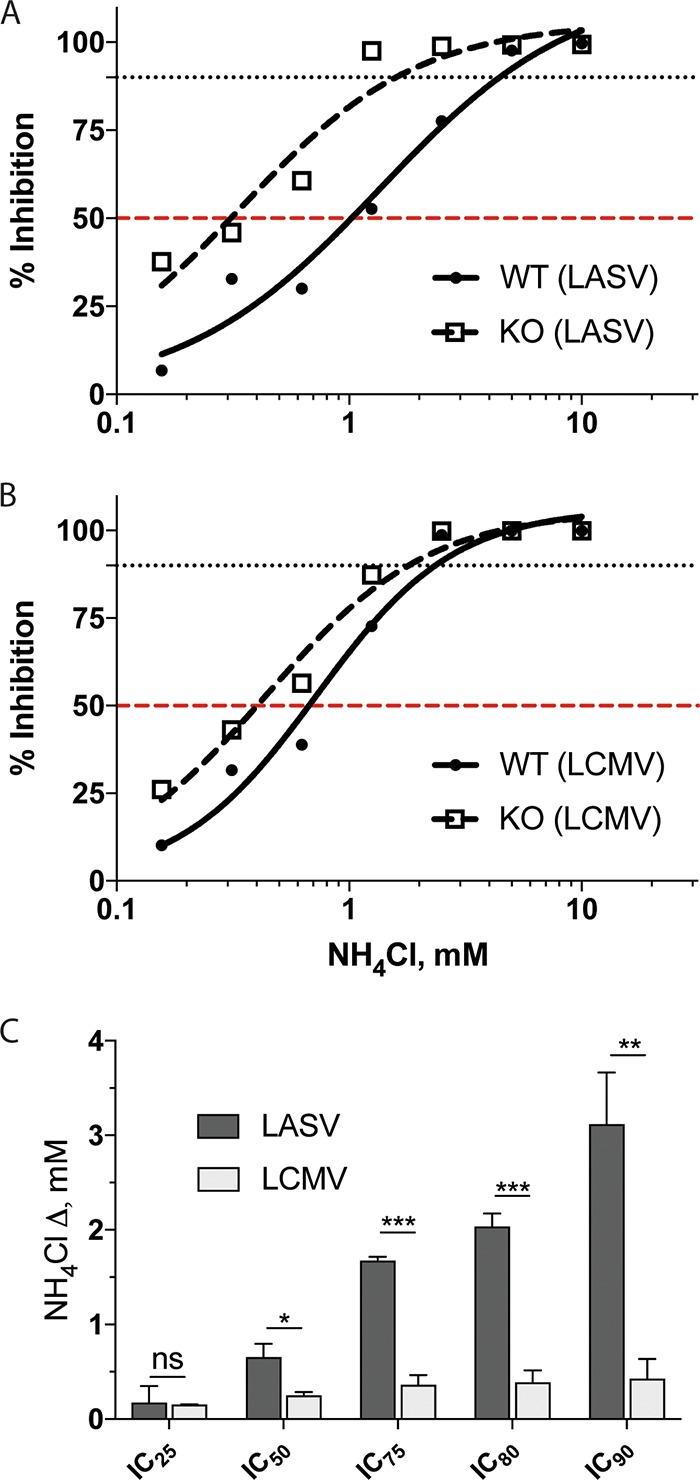
Dose responses of LASV and LCMV GPC-mediated infection to NH_4_Cl in cells ± Lamp1. WT (solid lines, filled circles) and KO (dashed lines, empty boxes) cells were pretreated with NH_4_Cl at the indicated concentrations. Cells were then infected in triplicate with (A) LASV or (B) LCMV MLV pseudoviruses (titer determined for ~75,000- to 100,000-RLU signal in mock-treated WT cells). At 24 hpi, cells were lysed and analyzed for firefly luciferase activity. Infection signal was normalized to mock-treated cells, converted to inhibition values, and fitted to a sigmoidal dose-response curve. 50% and 90% inhibition are indicated by red and black dashed lines, respectively. Data shown in panels A and B are from a single experiment that was performed two additional times with similar results. In panel C, the average differences between inhibitory NH_4_Cl concentrations for LASV and LCMV in WT and KO cells from the three experiments are shown (ΔmM = IC_WT_ − IC_KO_). See Table 1 for details. *, *P* < 0.05; **, *P* < 0.01; and ***, *P* < 0.001.

Using a visual syncytial assay, Jae et al. reported no LASV GPC-mediated fusion at pH 5.5 in Lamp1-deficient 293T cells (generated by TALEN [transcription activator-like effector nuclease]-mediated gene disruption); robust syncytium formation at the same pH was, however, observed in cells overexpressing a mutant Lamp1 directed to the cell surface (9). Earlier observations have documented an unusually low pH requirement for both LASV and LCMV GPC-mediated syncytium formation, with optimal activity at pH ≤4 (17, 18, 23). Lamp1 is progressively enriched in maturing endosomes and has been reported to be most abundant in late endosomes, where the pH range is ~4.5 to 5.5 (24). Coupling these three prior observations with our finding that LASV GPC-mediated entry and infection can occur (albeit at reduced efficiency) in Lamp1 KO cells, we postulated that by binding to LASV GPC (9, 11, 25, 26), Lamp1 promotes fusion at a higher (less acidic) pH. We describe three lines of experimentation to test this hypothesis (Fig. 4 to 7).

In the first set of experiments, we employed a highly sensitive split luciferase cell-cell fusion assay (27, 28) to rigorously assess the extent and pH dependence of LASV GPC-mediated cell-cell fusion in the presence and absence of Lamp1 at the cell surface over a range of pH values. In this experiment (diagrammed schematically in Fig. S2A in the supplemental material), one set of 293T cells expressed LASV or LCMV GPC and one-half of a split luciferase/GFP construct. This set was then cocultured with target 293T cells expressing the other half of the split luciferase/GFP construct and different levels of cell surface Lamp1: WT, Lamp1 KD, Lamp1 KO, or cells transiently overexpressing plasma membrane-directed Lamp1 (pmLamp1). The cocultures were then briefly exposed to buffers of defined pH, reneutralized, and assayed for luciferase activity after 1 h. The different levels of Lamp1 on the surface of the target cells, determined by flow cytometry, are shown in Fig. S2B. Note that pmLamp1 cells express at least 20-fold more Lamp1 at the cell surface than WT or KD cells, both of which have little to no detectable surface Lamp1.

10.1128/mBio.01818-17.2 FIG S2  Cell-cell fusion assay schematic. (A) Effector cells (left) are transfected to express either LASV or LCMV GPC and one-half of a dual split protein, DSP1 (“DSP” represents luciferase and GFP). Target cells (right) are transfected to express either DSP2 alone or DSP2 plus pmLamp1. After providing a luciferase substrate to effector cells, effector cells are lifted and overlaid onto the target cells, and the cocultured cells are then pulsed with pH-adjusted buffer to trigger GPC-mediated cell-cell fusion. Following reneutralization and a further 1-h incubation, the luminescence from the reconstituted luciferase reporter is recorded as an indicator of fusion. (B) The percentage of target cells with detectable Lamp1 at the surface was determined by flow cytometry. See Materials and Methods for detailed information. Download FIG S2, TIF file, 32.8 MB. Copyright © 2018 Hulseberg et al. 2018 Hulseberg et al. This content is distributed under the terms of the Creative Commons Attribution 4.0 International license.

We first compared the fusogenicity of LASV GPC-expressing cells by coculturing them with either pmLamp1 or WT cells and then briefly pulsing with pH-adjusted buffer to trigger fusion. The difference in LASV GPC-mediated fusion efficiency with target cells was evident at pH <6, where fusion with pmLamp1 cells was 1 to 2 log units higher than with WT cells (measured in increments of 0.5 pH unit). When fusion at all pH values was normalized to activity at pH 4.5, a prominent, upward pH shift in fusogenicity was seen with pmLamp1 cells (Fig. 4B). For example, fusion with WT cells at pH 5.0 was ~20% of that seen at pH 4.5, while fusion with pmLamp1 cells at the same pH was similar to that seen at pH 4.5. Even at pH 5.5, there was appreciable fusion (~40% of that seen at pH 4.5) with pmLamp1 cells, whereas only background levels of fusion were seen with WT cells. Notably, LASV GPC-mediated fusion with Lamp1-deficient KD and KO cells was not significantly different relative to WT cells (see Fig. S3 in the supplemental material), as expected, given the virtually undetectable levels of Lamp1 at the surfaces of KD and KO cells (Fig. S2B). To test the specificity of the Lamp1-dependent change in fusion pH, we assayed LCMV GPC-mediated fusion with WT and pmLamp1 cells. As expected, Lamp1 neither increased the extent nor altered the pH threshold of LCMV GPC-mediated fusion (Fig. 4C and D).

10.1128/mBio.01818-17.3 FIG S3  Levels of LASV GPC-mediated cell-cell fusion with WT cells or cells expressing limited (KD) or no (KO) Lamp1 are not significantly different. In panels A and C, triplicate measurements of luminescence show the extent of LASV GPC-mediated fusion with WT cells compared to either KD (A) or KO (C) cells. In panels B and D, the corresponding normalized pH dependence of fusion with either KD (B) or KO (D) cells is shown. Statistical significance of fusion efficiency with WT or Lamp1 KD or KO cells at pH 5 and 5.5 was assessed using an unpaired, two-tailed *t* test. ns, not significant. Download FIG S3, TIF file, 2.7 MB. Copyright © 2018 Hulseberg et al. 2018 Hulseberg et al. This content is distributed under the terms of the Creative Commons Attribution 4.0 International license.

As a complementary approach, we tested whether overexpression of pmLamp1 affects the fusion pH of intact, cell-bound pseudoviruses using a system that bypasses the normal endocytic pathway and forces virus fusion at the plasma membrane (29, 30). LASV GPC VSV pseudoviruses bearing a luciferase reporter were bound to WT or pmLamp1-expressing COS-7 cells in the cold for 1 h. Cells were then briefly exposed to a range of pH-adjusted buffers to trigger fusion before reneutralizing the media. Immediately following reneutralization, cells were treated with the lysosomotropic agent NH_4_Cl to inhibit acidification of endosomes and therefore block natural entry through the endocytic pathway. After 24 h, GPC-mediated LASV pseudovirus fusion with the plasma membrane was assessed by luciferase output. As seen in Fig. 5, in the absence of pmLamp1, a low level of fusion was observed at pH 5.0 and 5.5. (Forced fusion at the plasma membrane could not be reliably assessed at pH 4.5 due to severe cell loss.) In sharp contrast, strong fusion signals were observed at pH 5.0 and 5.5 with pmLamp1 cells. Thus, the more alkaline pH threshold for LASV GPC-mediated cell-cell fusion (Fig. 4) and virus-cell fusion (Fig. 5) strongly suggests that Lamp1 facilitates fusion of LASV particles in less acidic endosomes when Lamp1 is present than when Lamp1 is lacking.

### Lamp1 promotes LASV GPC-mediated entry in less acidic endosomes.

Lamp1 promotes both cell-cell fusion (Fig. 4) and pseudovirus-cell fusion (Fig. 5) at pH 5.0 to 5.5, while significant fusion in the absence of Lamp1 is only supported at pH ≤4.5. Thus, we postulated that in WT cells, LASV GPC-mediated entry occurs in endosomes that are less acidic than the endosomes from which LASV GPC directs fusion when Lamp1 is absent. If this were the case, then LASV should more adeptly infect WT cells than Lamp1 KO cells when the pH is raised with an inhibitor of endosomal acidification. In other words, LASV infection in Lamp1 KO cells should be more sensitive to the effects of NH_4_Cl than infection in WT (Lamp1-positive) cells. To test this hypothesis, we progressively raised the endosomal pH with increasing concentrations of NH_4_Cl to compare the effect on LASV GPC-mediated pseudovirus infection in WT versus Lamp1 KO cells. Since infection in Lamp1 KO cells is ~20% that seen in WT cells, we used two inputs (low and high) of titer-determined LASV MLV pseudoviruses to achieve a roughly equivalent infection signal in WT (low input) and Lamp1 KO (high input) cells. As seen in Fig. 6A, infection in Lamp1 KO cells was, indeed, more sensitive to the neutralizing effects of NH_4_Cl. Accordingly, a higher concentration of NH_4_Cl was needed to block LASV GPC-mediated infection in WT (Lamp1-positive) cells. (This effect was seen at both the low and high inputs of LASV pseudoviruses.) As expected, since LCMV does not require Lamp1 (Fig. 2B, D, and F and Fig. 5C and D), we did not see any difference in the NH_4_Cl sensitivity of LCMV infection in WT versus Lamp1 KO cells (Fig. 6B).

To more thoroughly evaluate the Lamp1-dependent change in sensitivity to NH_4_Cl (raising endosomal pH), we generated 8-point dose-response curves and determined inhibitory concentrations for both LASV and LCMV (Fig. 7; Table 1). Consistent with our earlier finding (Fig. 6), a greater difference in inhibitory concentrations between WT and KO cells was seen for LASV compared to LCMV GPC-mediated infections. In Table 1, we present inhibitory concentration values for the effects of NH_4_Cl on LASV and LCMV GPC-mediated infection in WT and KO cells. The differentials for these inhibitory concentrations (Table 1) are graphically compared in Fig. 7C, which clearly shows a pronounced change in sensitivity of LASV, but not LCMV, to NH_4_Cl in Lamp1 KO versus WT cells.

**TABLE 1:** Concentrations of NH_4_Cl needed to inhibit LASV and LCMV GPC-mediated infection in WT or KO cells

IC	Result (mM) for^a^:					
	LASV			LCMV		
	IC_WT_	IC_KO_	Δ = IC_WT_ − IC_KO_	IC_WT_	IC_KO_	Δ = IC_WT_ − IC_KO_
IC_25_	0.48 ± 0.08	0.30 ± 0.21	0.18 ± 0.14	0.32 ± 0.01	0.17 ± 0.01	0.15 ± 0.00
IC_50_	1.17 ± 0.15	0.51 ± 0.26	0.65 ± 0.12	0.64 ± 0.04	0.39 ± 0.02	0.25 ± 0.03
IC_75_	2.57 ± 0.31	0.89 ± 0.28	1.68 ± 0.03	1.24 ± 0.12	0.87 ± 0.04	0.36 ± 0.08
IC_80_	3.07 ± 0.39	1.03 ± 0.28	2.04 ± 0.11	1.45 ± 0.15	1.06 ± 0.05	0.39 ± 0.10
IC_90_	4.65 ± 0.70	1.53 ± 0.28	3.12 ± 0.45	2.16 ± 0.26	1.73 ± 0.09	0.43 ± 0.17

^a^ Data are the averages ± SD from three experiments. The differences between the WT and KO values for both LASV and LCMV are shown graphically in Fig. 7C.

Collectively, the results in Fig. 4 to 7 suggest that LASV GPC-mediated fusion and entry occur in less acidic endosomes when Lamp1 is present than when Lamp1 is absent, whereas LCMV fusion and entry occur in endosomes with the same approximate pH in cells containing or lacking endogenous Lamp1.

## DISCUSSION

**FIG 8:**
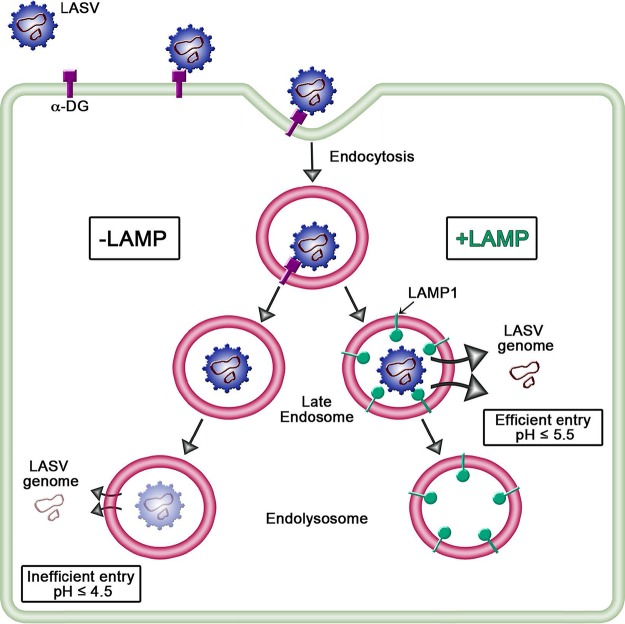
Model of LASV entry into cells ± Lamp1. After initial attachment to α-DG at the cell surface, LASV particles are internalized into compartments within the endocytic pathway. The proposed pathway for Lamp1-negative (KO) cells (left) indicates LASV GPC-mediated fusion and entry from highly acidic endosomes. In Lamp1-positive (WT) cells, the receptor switch to Lamp1 elevates the pH threshold for GPC-mediated fusion, ensuring efficient entry from a less acidic endosome. We further propose that entry in Lamp1-positive cells is more efficient because the particles avoid inactivation by extremely low pH and/or proteases within less hospitable, Lamp1-negative endosomes.

In the present study, we provide evidence that Lamp1 plays a significant, but not absolutely essential, role in LASV entry, and we further provide evidence for how Lamp1 promotes LASV entry. Our findings can be summarized as follows. (i) A robust (~85%) decrease in Lamp1 expression does not dampen the efficiency of LASV pseudovirus infection of 293T cells over a range of input multiplicities. (ii) Knockout of Lamp1 expression in 293T cells diminishes, but does not abolish, entry and infection (shown using three different sets of LASV pseudoviruses bearing different reporters [luciferase, GFP, and βlaM] as well as different viral cores [VSV and MLV]). (iii) LASV GPC-mediated fusion, evidenced in both cell-cell and pseudovirus-cell surface fusion assays, is markedly more active at a higher pH when Lamp1 is present. This suggested that LASV entry occurs in less acidic endosomes when they contain Lamp1. (iv) Indeed, LASV pseudovirus infection is more efficient in WT (Lamp1-positive) cells treated with a given amount of an inhibitor of endosomal acidification than in KO (Lamp1-negative) cells. We propose that by promoting fusion and entry in less acidic endosomes, Lamp1 increases the overall efficiency of LASV entry and infection (Fig. 8).

It was initially curious to us that the strong reduction in Lamp1 in the shRNA-mediated KD cells failed to affect even a modest decrease in LASV infection efficiency. However, given the ubiquitous expression and high abundance of Lamp1, which accounts for ~0.1% of total cellular protein and has been estimated to reach ~2 million Lamp1 molecules per cell (31, 32), it is likely that the remaining Lamp1 in these KD cells is a surfeit to support LASV GPC-mediated pseudovirus infection, even at the highest inputs of pseudovirus tested. Furthermore, although Lamp1 reaches peak enrichment within acidic late endosomes/lysosomes, LASV GPC-Lamp1 binding is biochemically feasible at pH ≤6.5 (9). Thus, the receptor switch from α-DG to Lamp1 (9) might be handily supported within earlier endosomes, despite relatively light carriage of Lamp1 in these maturing compartments (33, 34).

In our system, lack of Lamp1 did not confer the full resistance to LASV infection expected from loss of an absolutely required receptor. One possibility to explain this would be compensatory interactions with another endosomal protein(s). A leading candidate, Lamp2, is not likely to play such a role: it did not emerge in the screen for pro-LASV factors, did not rescue infection in Lamp1 KO cells, and does not appear to physically interact with LASV GPC (9). Interestingly, the endosomally concentrated tetraspanin CD63 was recently identified as promoting fusion and infection by the Old World arenavirus, Lujo virus (LUJV) (35). It would therefore be interesting to know whether CD63 can (partially) support LASV entry in cells lacking Lamp1. Another possibility is that the level of resistance of LASV infection in Lamp1 KO cells varies among cell types. We note, however, that studies with WT and Lamp1^−/−^ mice also intimated an important, albeit perhaps not absolutely essential role for Lamp1: at 6 days postinfection, Lassa virus titers remained high in WT tissues but fell below the detection limit in Lamp1^−/−^ tissues; however, at 3 days postinfection, comparable levels of virus were found in serum and LASV was also detected in spleen in Lamp1^−/−^ mice. This suggests that by day 6, inefficient viral entry in Lamp1^−/−^ mice may have afforded an opportunity for the immune system to clear the infection. In the context of a physiologic Lassa infection in a homozygous Lamp1-deficient, but otherwise susceptible and immunocompetent host, perhaps a reduced number of Lassa virus particles escaping from late, highly acidified endocytic compartments allows for the establishment, but not sustainment, of infection.

Before the importance of Lamp1 in LASV entry was realized (9), several groups concluded (from cell-cell fusion-based evidence) that LASV GPC-mediated fusion occurs under remarkably acidic (pH ≤4.5) conditions, perhaps even within lysosomes (17, 18). Our work (Fig. 4 and 5) and that of Jae et al. (9) indicate, however, that robust LASV GPC-mediated fusion can occur at pH 5.5 if Lamp1 is present. Moreover, we provide evidence (Fig. 6 and 7) that LASV GPC-mediated entry occurs in less acidic endosomes in Lamp1-positive versus Lamp1-negative endosomes, as modeled in Fig. 8. A corollary is that in the absence of Lamp1, LASV must traffic to more acidic, and potentially more proteolytic, endosomes, which may inactivate significant numbers of LASV particles before they are able to fuse. We further propose that by binding to LASV GPC, Lamp1 promotes a critical fusion-inducing conformational change at a higher pH than when Lamp1 is absent. Future experiments are needed to test this hypothesis and, if correct, to elucidate the specific change involved, whether dissociation of GP1 from GP2 or refolding of GP2 to prehairpin or hairpin conformations (14).

The question arises as to whether other Old World arenaviruses employ intracellular (endosomal) receptors. A second example is likely LUJV. As mentioned above, CD63 enhances fusion and entry by LUJV. However, unlike for LASV GPC and Lamp1, a binding interaction has not yet been observed between LUJV GPC and CD63 (35). What about LCMV, the prototypical Old World arenavirus? LCMV GPC-mediated infection, entry, and fusion were not affected by the absence of Lamp1 (Fig. 1 to 4, 6, and 7) (9), and loss of CD63 did not impair LCMV GPC-mediated infection (35). Moreover, consistent with the observed pH dependence of LCMV fusion being remarkably low (optimal at pH ≤4.5) regardless of the presence or absence of Lamp1, we found that LCMV infection (in WT cells) is considerably more sensitive to NH_4_Cl than LASV infection; it is, in fact, quite similar to the sensitivity of LASV infection in cells lacking Lamp1 in endosomes (Fig. 6B). While it certainly remains possible that LCMV employs a proviral endosomal fusion factor, the aforementioned collective observations suggest that this may not be the case. If so, it is possible that LCMV GPC is better able to tolerate the harsher conditions within more acidic endosomes than LASV (and LUJV) and therefore may undergo low-pH-dependent fusion activation unassisted by endosomal receptors. Future experiments will be needed to test this hypothesis and to fully assess which arenaviruses do (9, 35) and which, if any, do not employ assisted fusion in endosomes.

## MATERIALS AND METHODS

### Cells.

HEK 293T/17 cells (human embryonic kidney fibroblasts; ATCC CRL-11268 via University of Virginia Tissue Culture Facility), BHK21 cells (baby hamster kidney fibroblasts; ATCC CCL-10 [a kind gift from James Casanova at the University of Virginia]), and COS-7 cells (African Green monkey fibroblasts; ATCC CRL-1651 [a kind gift from Douglas DeSimone at the University of Virginia]) were maintained at 37°C with 5% CO_2_ in growth medium: high-glucose Dulbecco’s modified eagle medium (DMEM) supplemented with 1% l-glutamine, 1% sodium pyruvate, 1% antibiotic/antimycotic, and 10% supplemented calf serum (SCS; Hyclone, GE Healthcare Bio-Sciences).

### shRNA knockdown of Lamp1.

Validated shRNA against human Lamp1 (5′ CCGGTGCTGCTGCCTTCTCAGTGAACTACTCGAGTAGTTCACTGAGAAGGCAGCATTTTT 3′) in pLKO.1-puro vector was purchased from Sigma-Aldrich (clone TRCN0000029268). To produce lentiviruses, parental 293T cells (7 × 10^5^ cells per 6-cm^2^ tissue culture dish) were transfected with 1 μg pLKO.1 shRNA plasmid, 900 ng psPAX2 packaging plasmid, and 100 ng pDM2.G envelope plasmid using FuGENE 6 transfection reagent. The following morning, medium was harvested and centrifuged at 1,000 × *g* for 5 min to remove cellular debris, and the clarified, lentivirus-containing medium was filtered through 0.45-μm-pore filters. 293T cells (~40% confluent) were transduced with the lentivirus using 6 μg/ml Polybrene. At 96 hpi, cells were split and transduced cells were selected with 3 μg/ml of puromycin. The extent of Lamp1 knockdown was determined by Western blotting of whole-cell lysates and visualized using the Odyssey infrared imaging system (Licor). Protein quantification was performed using ImageJ software.

### CRISPR/Cas9-mediated knockout of Lamp1.

Lamp1 knockout (KO) cell lines were generated by CRISPR/Cas9-mediated genome editing. A guide RNA (gRNA) targeting the first exon of Lamp1 was selected using Thermo Fisher’s GeneArt CRISPR Design Tool. Primers for both strands covering the cleavage site (F, 5′ caccGAACGGGACCGCGTGCATAA 3′; R, 5′ aaacTTATGCACGCGGTCCCGTTC 3′ [lowercase letters indicate the complementary BbsI overhangs]) were annealed to each other to make a double-stranded oligonucleotide that was then cloned into the BbsI site of the pX330-U6-Chimeric_BB-CBh-hSpCas9 vector, which has both a gRNA scaffold site and Cas9 (the plasmid [Addgene plasmid 42230] was a kind gift from Mazhar Adli at the University of Virginia and Feng Zhang) (36, 37). After sequencing to confirm correct insertion of the gRNA, pX330-U6 and an enhanced green fluorescent protein expression plasmid (to assess transfection efficiency) were cotransfected into 293T cells using Lipofectamine 2000. Lamp1 expression in transfected and untransfected populations was crudely compared by Western blotting. Cells with the gRNA treatment resulting in the lowest Lamp1 expression were stained with Lamp1 antibody (H4A3 from the Developmental Studies Hybridoma Bank) and Alexa Fluor 488 and subjected to negative selection for no/low surface Lamp1 expression via fluorescence-activated cell sorter (FACS). After expansion of singly sorted cells, clonal cell lines were permeabilized with 0.05% saponin, stained with Lamp1 antibody, and screened for null Lamp1 expression by in-cell Western (ICW) assay on a 96-well format as previously described (38). From the Lamp1-negative clonal cell lines identified by ICW, eight clones were selected for further confirmation by traditional Western blot. To confirm gene disruption near the PAM site, a fragment of genomic DNA from parental (WT) cells and two of the clonal Lamp1 KO lines was amplified (F, 5′ ACCCCAGCCTGGCGACAGTGAGACTCC 3′; R, 5′ ATGGCACATGACAGCGCAGGTTACTGACA 3′) and cloned into a TOPO vector, and the region of interest was then sequenced to confirm gene disruption (5′ CCGTCTTCCCTGGAATTGACAGGCCTCAT 3′).

### Generation of plasma membrane Lamp1.

To transiently overexpress Lamp1 at the plasma membrane (pmLamp1), Agilent’s QuikChange II protocol was followed to delete the codon for Ala^384^ in the Lamp1 gene (pRK5-LAMP1-FLAG plasmid [Addgene plasmid 71868] was a gift from David Sabatini) (39, 40). The sequence of the forward primer was 5′ GTCGGCAGGAAGAGGAGTCACGGCTACCAGACTATCTAGGCGGCCGCGATC 3′, and that of the reverse primer was 5′ GATCGCGGCCGCCTAGATAGTCTGGTAGCCGTGACTCCTCTTCCTGCCGAC 3′. The underlined residues represent the flanking residues around the deleted codon for alanine.

### Viruses.

To produce MLV pseudovirus particles (with either luciferase, Gag-βlaM, or both reporters), 293T cells in 10-cm^2^ dishes were grown in DMEM containing 10% SCS to 80% confluence and then transfected with 6 μg of total DNA with the following plasmids at a 2:1:1:1 ratio: pTG-luc (a kind gift from both Jean Dubuisson at the Centre National de la Recherche Scientifique in Lille via Gary Whittaker at Cornell University), pCMV gag-pol (from Jean Millet at Cornell University and Jean Dubuisson), Gag-βlaM (produced by James Simmons), and glycoprotein (LASV Josiah strain GPC in pCMV from Gregory Melikyan at Emory University; LCMV Armstrong strain GPC in pCMV from Jack Nunberg at University of Montana and Juan de la Torre at Scripps Institute). At 48 h posttransfection (hpt), virus-containing medium was harvested, clarified by low-speed centrifugation, and filtered through 0.45-μm-pore filters. Pseudoviruses were stored on ice, and their titers were determined to achieve desired functional ranges by either luciferase infection assay or βlaM entry assay. After titration, pseudovirus stocks were stored at −80°C.

To produce VSV-luciferase pseudoviruses, 5 × 10^5^ BHK21 cells were seeded (40 10-cm^2^ dishes) in DMEM containing 10% SCS. Cells were transfected with 12 μg of plasmid expressing LASV GPC, LCMV GPC, or no GPC, using polyethylenimine (PEI). The following day, cells were infected for 1 h at 37°C with VSV-G helper virus expressing *Renilla* luciferase (diluted in serum-free medium). After infection, cells were thoroughly washed with cold phosphate-buffered saline (PBS) and incubated overnight in complete DMEM. Supernatants containing pseudoviruses were collected and concentrated using a Viva-Spin 20 (300-kDa molecular mass cutoff) and then pelleted through a 20% sucrose–HM (20 mM HEPES, 20 mM MES [morpholineethanesulfonic acid], 130 mM NaCl, pH 7.4) cushion. The pellet was resuspended in sterile 10% sucrose–HM.

To produce VSV-G luciferase helper virus, 5 × 10^5^ BHK21 cells were seeded (5 10-cm^2^ dishes) in DMEM containing 10% SCS. Cells were transfected with 12 μg of plasmid expressing VSV-G using PEI. The following day, cells were infected for 1 h at 37°C with VSV-luciferase plaques in serum-free DMEM. After infection, cells were thoroughly washed with cold PBS and incubated overnight in complete DMEM at 37°C. Supernatants containing helper viruses were collected and stored at −80°C.

### Infection assay.

293T cells (WT, Lamp1 KD, or Lamp1 KO) in DMEM containing 10% SCS were seeded onto fibronectin-coated white 96-well plates (3 × 10^4^ cells/well). The following morning, cells were infected with an input of pseudovirus titer determined to achieve a target signal range and incubated at 37°C. At 48 h postinfection (hpi), cells infected with luciferase pseudoviruses were washed with PBS and lysed with Britelite reagent (PerkinElmer), which also contains firefly luciferase substrate, and incubated for 10 min at room temperature while shaking before measuring luminescent output on a Promega GloMax luminometer. For assays using GFP-expressing pseudovirus infections, at 48 hpi, cells were washed, fixed, and analyzed for fluorescence by flow cytometry. For assays involving inhibition of infection by NH_4_Cl (41), cells were pretreated with drug diluted in Opti-MEM I (OMEM) 1 h before infection with pseudovirus, as described elsewhere. Interpolated inhibitory concentrations and statistical analysis of all data were performed using GraphPad Prism 7 (GraphPad Software, Inc.).

### Entry assay.

To assess GPC-mediated entry, 293T cells grown in DMEM containing 10% SCS were seeded onto fibronectin-coated transparent 96-well plates. After 18 to 24 h, a titer-determined input of βlaM pseudoviruses was diluted in OMEM and bound to cells by spinfection at 250 × *g* for 1 h at 4°C. Cells were incubated at 37°C for 3 h before adding βlaM substrate (CCF2-AM; Invitrogen) and allowing an additional 1 h of incubation at 37°C. Cells were then washed with PBS and allowed to incubate overnight at room temperature in loading buffer (phenol red-free DMEM, 5 mM probenecid, 2 mM l-glutamine, 25 mM HEPES, 200 nM bafilomycin, 10% fetal bovine serum [FBS]). The following day, cells were washed with PBS, fixed in 4% paraformaldehyde, and analyzed for virus entry by flow cytometry using a BD FACSCalibur or on a BioTek Cytation3 plate reader.

### Cell-cell fusion assay.

Effector populations of 293T cells (i.e., cells expressing LASV or LCMV GPC) were seeded onto 6-well plates (3.75 × 10^5^ cells/well). Receptor cell populations (i.e., cells representing Lamp1 phenotypes [pmLamp, WT, Lamp1 KD or KO cells]) were seeded onto fibronectin-coated white 96-well plates (3 × 10^4^ cells/well). The following morning, effector cells were transfected with 1 μg/well of either LASV or LCMV GPC plasmids and an equivalent amount of DSP_1−7_ plasmid (a kind gift from Naoyuki Kondo) (28); receptor cells were transfected with 33 ng/well of pRK-pmLamp1 vector (when overexpressing Lamp1 at the plasma membrane) and an equivalent amount of DSP_8−11_ plasmid. Lipofectamine 2000 was used for all transfections. At 24 hpt, effector cell medium was replaced with fresh DMEM containing 60 μM EnduRen luciferase substrate (Promega). After incubation for 2 h at 37°C, effector cells were rinsed with PBS, detached with 0.05% trypsin-EDTA, and overlaid onto receptor cells (1.5 × 10^5^ cells/well). The mixed cell populations were allowed to settle for 3 h at 37°C before pH pulsing the cells with HMS buffer (100 mM NaCl, 15 mM HEPES, 15 mM succinate, 15 mM MES, 2 mg/ml glucose) adjusted to the appropriate pH for 5 min at 37°C. The pH was then reneutralized with 20 mM HEPES in DMEM, and cells were returned to 37°C for 1 h before recording luminescence on a Promega GloMax luminometer.

### Forced fusion at the plasma membrane.

COS-7 cells were seeded on a 6-well plate to reach 60% confluence the day of transfection. Cells were transfected using Lipofectamine 2000 with 0.6 μg of pmLamp1 DNA and 1 μg of firefly luciferase DNA according the manufacturer’s instructions. pmLamp1-transfected or firefly luciferase-only transfected COS-7 cells were seeded at a density of 15,000 cells/well on a fibronectin-coated white 96-well plate. The next day, cells were cooled on ice for 15 min. LASV VSV-luciferase (*Renilla*) pseudoviruses, the titer of which had been determined to reach a target signal under control conditions, were added to cells in sextuplicate in serum-free DMEM at pH 6.5 and bound to the cells by centrifugation (250 × *g*, 1 h, 4°C). Cells were returned to ice and washed once with cold PBS. To promote fusion at the plasma membrane, a pH pulse was applied for 5 min at 37°C in prewarmed HMS buffer at different pH values (7.0, 6.0, 5.5, and 5.0). Cells were returned to ice, and complete DMEM containing 40 mM NH_4_Cl (to block virus entry) was added. Sixteen hours later, luciferase activities were measured using the Dual-Glo luciferase assay system (Promega) according to the manufacturer’s instructions. Viral fusion with the plasma membrane was assessed by using a ratio of *Renilla* luciferase activity (an indicator for virus replication) over firefly luciferase activity (to account for the number of cells).
