# Supplementary figures and images for: Lamp1 Increases the Efficiency of Lassa Virus Infection by Promoting Fusion in Less Acidic Endosomal Compartments
Source: mBio. 2018 Jan 2;9(1):e01818-17. doi: 10.1128/mBio.01818-17 (PMC5750398; doi:10.1128/mBio.01818-17)

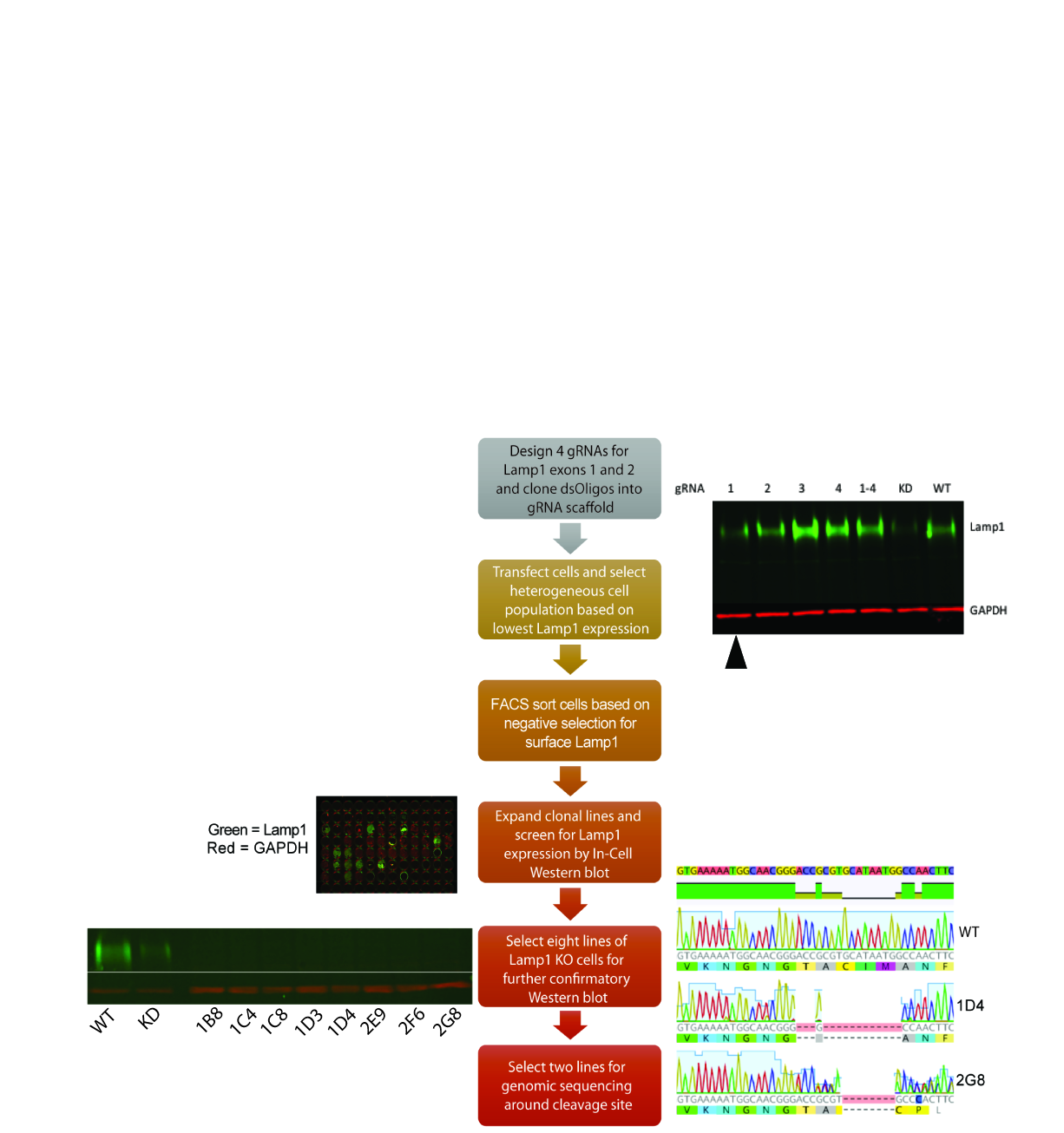

Supplement: FIG S1 [file mbo001183660sf1.tif]

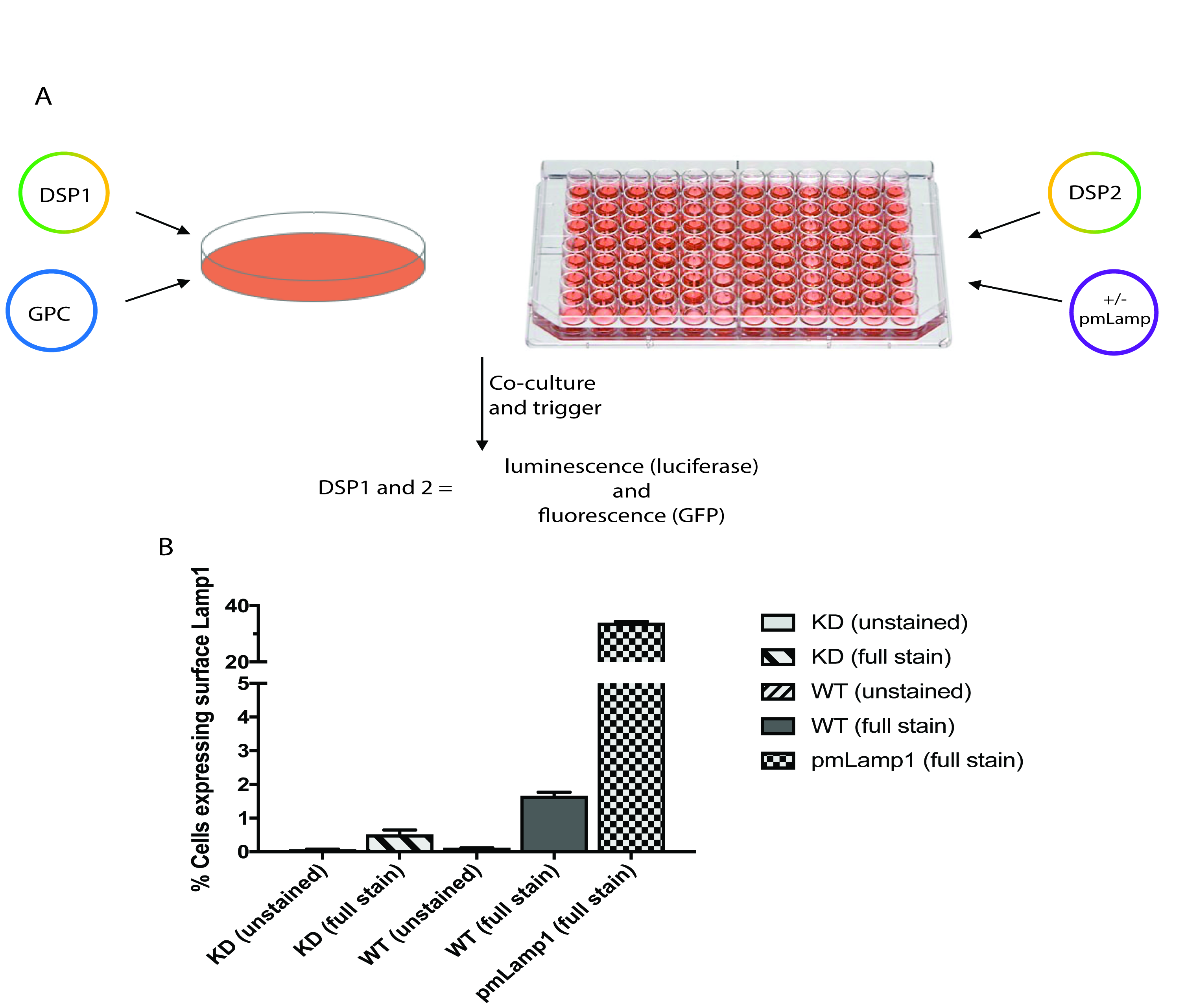

Supplement: FIG S2 [file mbo001183660sf2.tif]

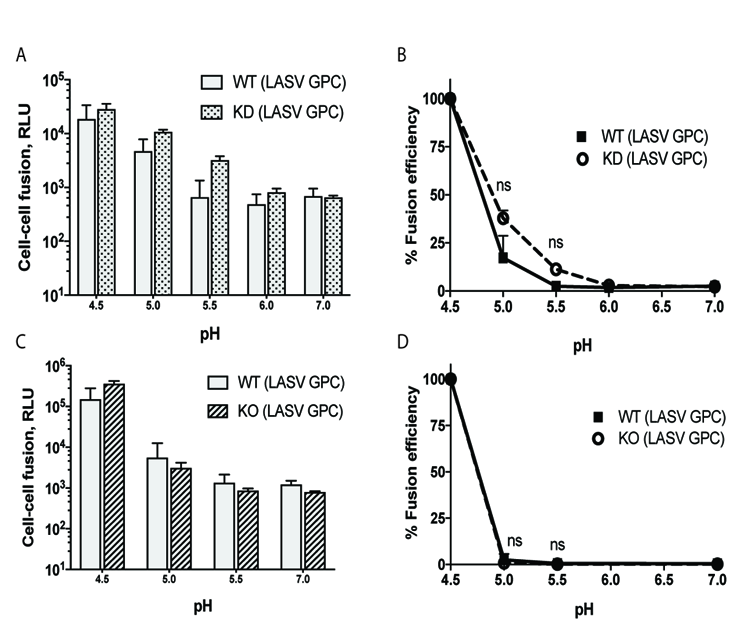

Supplement: FIG S3 [file mbo001183660sf3.tif]
